# Optimizing Surgical Approaches for Patients with Inherited Factor VII Deficiency

**DOI:** 10.1055/a-2778-4440

**Published:** 2026-01-06

**Authors:** Pablo García-Jaén, José Manuel Martín de Bustamante, Ana Mendoza-Martínez, Sara Galván-Platas, Rafael Monleón-Gil, Karla Susana Calzadilla-Román, Celia Nicolás-Boluda, Beatriz Rey-Bua, Carlos Puerta-Vázquez, Evelyn Zapata-Tapia, María Cortés-Rodríguez, Ana Hortal, Violeta Martínez-Robles, María José Cebeira-Moro, Covadonga García-Díaz, Agustín Rodríguez-Alén, Carlos Aguilar-Franco, Shally Marcellini, Elena María Fernández-Fontecha, Julio Dávila-Valls, Sandra Valle-Herrero, Rocío Benito, Nuria Bermejo, José Manuel Calvo-Villas, María Teresa Álvarez-Román, José Ramón González-Porras, José María Bastida

**Affiliations:** 1 Servicio de Hematología, Complejo Asistencial Universitario de Salamanca (CAUSA), Instituto de Investigación Biomédica de Salamanca (IBSAL), Universidad de Salamanca (USAL), Spain; 2 Servicio de Hematología, Hospital Universitario La Paz, Madrid, Spain; 3 Servicio de Hematología, Hospital Universitario Miguel Servet, Zaragoza, Spain; 4 Servicio de Hematología, Hospital San Pedro de Alcántara, Cáceres, Spain; 5 Servicio de Oncología Radioterápica, Complejo Asistencial Universitario de Salamanca (CAUSA), Spain; 6 Departamento de Estadística, Universidad de Salamanca (USAL), Spain; 7 Servicio de Pediatría, Gerencia de Atención Primaria de Salamanca, Spain; 8 Servicio de Hematología, Hospital Universitario de León, Spain; 9 Servicio de Hematología, Hospital Clínico Universitario de Valladolid, Spain; 10 Servicio de Hematología, Hospital Universitario de Burgos, Spain; 11 Servicio de Hematología, Hospital Universitario de Toledo, Spain; 12 Servicio de Hematología, Hospital Santa Bárbara de Soria, Spain; 13 Servicio de Hematología, Complejo Asistencial de Segovia, Spain; 14 Servicio de Hematología, Hospital Universitario Río Hortega, Valladolid, Spain; 15 Servicio de Hematología, Hospital Nuestra Señora de Sonsoles, Ávila, Spain; 16 Servicio de Hematología, Hospital Virgen de la Concha, Zamora, Spain; 17 Centro de Investigación del Cáncer (USAL-CSIC), IBSAL, IMCC, USAL, Salamanca, Spain

**Keywords:** factor VII, surgery, inherited, rare bleeding disorders

## Abstract

**Background:**

Inherited factor VII deficiency (FVIID) presents a highly variable bleeding phenotype. The weak correlation between plasma FVII levels (FVII:C) and bleeding severity results in diverse management strategies and complicates surgical decision-making.

**Objectives:**

To describe surgical management and bleeding outcomes in patients with FVIID, and to identify key decision-making variables and predictors of surgical bleeding.

**Patients/Methods:**

We conducted a multicenter, retrospective study of 380 surgeries performed in 215 patients with FVIID. Patients were classified by FVII:C levels as mild, moderate, or severe deficiency. Bleeding score (BS) was defined according to ISTH-BAT. Surgeries were categorized as low-moderate risk (LR) or high risk (HR) for bleeding. A decision-tree simulation was performed.

**Results:**

Most patients had mild FVIID (76%), and 68% of surgeries were classified as LR. Prophylactic treatment with tranexamic acid (TA) and/or rFVIIa was administered in 42.8% of LR and 62.8% of HR surgeries. Prophylaxis was given to 73.9% of moderate/severe and 41% of mild FVIID patients, especially for HR procedures. FVII:C levels and surgical bleeding risk were key factors that influenced the selection of treatment. The overall bleeding rate was 3.1% (HR: 9%; LR: 0.4%). Most bleeding events occurred in mild FVIID patients with BS ≥3. Our algorithm recommends hemostatic treatment for all moderate/severe, and for mild patients HR surgeries and LR procedures when BS is ≥3.

**Conclusion:**

FVII:C levels and surgery type influence prophylactic hemostatic treatment strategies. Patients with mild FVIID, higher BS, and no hemostatic treatment had a greater risk of bleeding. Bleeding score and procedural risk were identified as predictors of surgical bleeding.

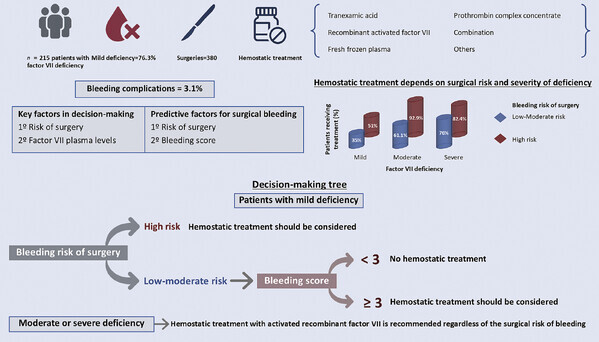

## Introduction

Inherited factor VII deficiency (FVIID) is the most common rare bleeding disorder (RBD), affecting approximately 1 in 500,000 individuals. It is caused by pathogenic variants in the *F7* gene and typically follows an autosomal recessive inheritance pattern. 1 Patients with FVIID exhibit a wide range of bleeding symptoms. These can vary from being asymptomatic or showing mild mucocutaneous bleeding to experiencing severe cases, such as spontaneous intracranial bleeding. 1 2 3 The most common symptoms include epistaxis and heavy menstrual bleeding. 4 5 6 Bleeding episodes in these patients can occur spontaneously or may be triggered by trauma or surgical procedures. 1 2 3 The presence of asymptomatic individuals and the absence of medical consultation in mild bleeding contribute to an underestimation of its actual prevalence. 7 8 Despite being a coagulation factor deficiency, cases of venous thrombosis have been reported in association with FVIID. 9 To classify the severity of FVIID, clinical and biological parameters are used. 10 11 The Bleeding Assessment Tool, developed by the International Society of Thrombosis and Hemostasis (ISTH-BAT), is commonly used to measure bleeding phenotype in patients with RBD, but it was not specifically designed for it. 12 Recently, more specific scales, such as the RBD-BAT score, have been developed to provide a better quantification of bleeding in these patients. 13 14 However, several studies have shown a weak correlation between plasma levels of factor VII (FVII:C, IU/dL) and the severity of clinical bleeding. 15 16 17 In that context, it has been demonstrated that isolated biological tests are not reliable for predicting bleeding risk in patients with inherited coagulation disorders under non-surgical conditions. 16 A combination of clinical phenotype and FVII:C has exhibited a stronger capacity to predict bleeding episodes. 18 One of the biggest challenges in managing surgical care for patients with FVIID is determining the appropriate treatment strategies. 19 20 21 22 These options include non-replacement, such as tranexamic acid (TA), and replacement therapies, with recombinant activated factor VII (rFVIIa), which is the most commonly used. 19 20 21 22 23 24 Most recommendations for surgical management are based on FVII:C. 25 26 Although some authors have emphasized the importance of clinical variables, there are not many studies that have successfully identified key factors influencing decision-making and predicting surgical bleeding. 25 Despite the availability of replacement therapies such as rFVIIa, there is a lack of standardized periprocedural management protocols, making surgical decision-making particularly challenging in these patients. Thus, the objectives of our study are to describe surgical management and perioperative hemostatic outcomes in patients with FVIID and to identify key decision-making parameters and potential predictors of surgical bleeding.

## Methods

### Patient Inclusion and Data Collection

We conducted a national, multicentric, retrospective, and descriptive study including 303 patients with inherited FVIID diagnosed between 2016 and 2024. Patients of all ages were included. Individuals with other coagulopathies (such as acquired or combined deficiencies), or those receiving anticoagulants, were excluded. We defined FVIID as having a FVII:C of less than 60 IU/dL. Thresholds for FVII:C by the European Network of Rare Bleeding Disorders (EN-RBD) classify deficiency as mild (>20 IU/dL), moderate (10–20 IU/dL), and severe (<10 IU/dL). 10 FVII:C was measured using a one-stage coagulation assay with recombinant human thromboplastin. Two platforms were used: the Sysmex CS-5100 analyzer (Sysmex Corporation, Kobe, Japan) with lyophilized normal plasma as calibrator (Siemens Healthineers, Marburg, Germany), and the Werfen ACL TOP 550 CTS analyzer (Werfen-Instrumentation Laboratory Company, Bedford, USA), using recombinant human tissue factor and FVII-immunodepleted human plasma, calibrated with lyophilized human plasma from the same manufacturer. Data collection was performed by trained hemostasis specialists through standardized personal interviews. Surgical history was gathered from data available at each center, which included information on bleeding complications and the incidence of thrombotic events within 30 days. The bleeding score (BS) was assessed by the ISTH-BAT to evaluate the bleeding phenotype. 12 Additionally, surgical procedures were categorized as low-moderate (LR) or high risk (HR) based on a combination of recommendations from the ISTH, WFH, and NICE guidelines. 27 28 29 According to the ISTH recommendations, surgical bleeding complications were graded, and hemostatic effectiveness was evaluated. 30 31 Major surgical bleeding was defined as any of the following: fatal bleeding; symptomatic bleeding in a critical area or organ; extrasurgical site bleeding resulting in a hemoglobin level decrease of at least 2 g/dL or requiring transfusion of two or more units of whole blood or red cells; surgical bleeding necessitating a second intervention; unexpected and prolonged surgical site bleeding large enough to cause hemodynamic instability. This study was conducted following the Helsinki Declaration and received approval from the Ethics Committees of the Instituto de Investigación Biomédica de Salamanca (IBSAL), Salamanca, Spain (reference PI 24/01458), and from each local ethics committee. All patients and/or family members gave written informed consent.

### Statistical Analysis

The associations between variables were analyzed using Pearson's correlation (r), Fisher's exact test, Chi-squared test, Mann-Whitney U, or ANOVA, depending on the type of data. Continuous variables were presented as median values and interquartile ranges (IQR: Q1–Q3). The strength of the correlation between BS and FVII:C was evaluated using Pearson's correlation, with the following thresholds: r < 0.100, very weak or no correlation; r = 0.100 to 0.299, weak correlation; r = 0.300 to 0.499, moderate correlation; r = 0.500 to 0.699, strong correlation; r ≥0.700, very strong correlation. Both univariate and multivariate analyses were performed to identify factors influencing hemostatic treatment decision-making. Based on expert clinical judgment, the following clinical and biological variables were analyzed for their potential relevance to decision-making: factor VII plasma levels, bleeding risk of surgery, bleeding score, surgical site, sex, and age at surgery. The Wald statistic was used to quantify the specific statistical weight of each variable. A decision-making tree simulation was performed using the CHAID (Chi-squared Automatic Interaction Detector) method. A *p* -value <0.05 was established to determine statistical significance. IBM SPSS Statistics v28.0.1.1(14) and JAMOVI v2.3 were used for statistical analysis.

## Results

### Patients and Procedures

The study included 303 patients, 49.5% of whom were females, with a median age of 32 years (IQR 20–53), with FVIID which was classified as mild in 236 patients (78%), moderate in 43 patients (14%), and severe in 24 patients (8%). The median BS was 0 (0–14); 113 patients (37%) reported a history of previous bleeding. The most common types of bleeding were epistaxis (20%) and mucocutaneous bleeding (16%). Among the women in the study, 23% reported experiencing heavy menstrual bleeding. There was an inverse weak correlation between BS and F:VIIC (Pearson's r = −0.297, *p* < 0.001). When the sample was divided into subgroups, we found a very weak or no correlation in patients with mild (r = −0.016) and moderate (r = −0.033) FVIID, whereas in patients with severe FVIID, the correlation was moderate (r = −0.322). The primary reason for diagnosis, noted in 47% of cases, was an alteration in routine pre-surgical coagulation tests, specifically the prothrombin time (PT), and one-third of the patients reported a family history of FVIID. A total of 215 (71%) patients, 43.7% of whom were females, with a median age of 34 years (IQR 16–53), underwent 380 surgical procedures. Surgeries were categorized as LR in 259 (68%) cases and HR in 121 (32%). The most common LR surgeries included various operations, with the most frequent being the repair of abdominal hernias and appendectomies. In contrast, the most frequent HR surgeries involved otorhinolaryngological procedures, such as tonsillectomies, and major traumatological surgeries, including total hip replacements. Perioperative hemostatic treatment was administered in 62.8% of HR surgeries and 42.8% of LR procedures ( Table 1 ).

**Table 1:** Baseline characteristics of the 215 inherited factor VII deficiency patients who underwent surgery

Age at the time of surgery (y), median (IQR)	34 (16–53)
Sex, *n* (%)	
Female	94 (43.7%)
Male	121 (56.3%)
Preoperative bleeding score (ISTH BAT), median (IQR)	0 (0–14)
FVII plasma level (IU/dL), median (IQR)	32.4 (20–42)
FVII:C plasma level, *n* (%)	
Severe (<10)	18 (8.4%)
Moderate (10–20)	33 (15.3%)
Mild (>20)	164 (76.3%)
Total surgeries, *n* (%)	380 (100%)
Low bleeding risk	259/380 (68%)
Miscellaneous surgery	43 (16.6%)
Digestive surgery	34 (13.1%)
Otorhinolaryngological surgery	29 (11.2%)
Urologic surgery	29 (11.2%)
Traumatological/Orthopedic surgery	28 (10.8%)
Single tooth extraction	24 (9.3%)
Ophthalmological surgery	21 (8.1%)
Dermatologic surgery	21 (8.1%)
Gynecologic surgery	20 (7.7%)
Vascular surgery	8 (3.1%)
Cardiac surgery	2 (0.8%)
High bleeding risk	121/380 (32%)
Otorhinolaryngological surgery	24 (19.9%)
Traumatological/Orthopedic surgery	23 (19%)
Urologic surgery	20 (16.5%)
Miscellaneous surgery	16 (13.2%)
Digestive surgery	16 (13.2%)
Gynecologic surgery	9 (7.4%)
Multiple tooth extraction	4 (3.3%)
Neurosurgery	3 (2.5%)
Cardiac surgery	3 (2.5%)
Vascular surgery	3 (2.5%)
Perioperative hemostatic treatment in low bleeding risk surgery, *n* (%)	111/259 (42.8%)
Tranexamic acid	55 (49.6%)
rFVIIa	40 (36%)
Fresh frozen plasma	2 (1.8%)
Combination (rFVIIa + TA)	13 (11.7%)
Other	1 (0.9%)
Perioperative hemostatic treatment in high bleeding risk surgery, *n* (%)	76/121 (62.8%)
rFVIIa	33 (43.5%)
Tranexamic acid	28 (36.8%)
Prothrombin complex concentrate	1 (1.3%)
Combination (rFVIIa + TA)	13 (17.1%)
Other	1 (1.3%)

Abbreviations: FVII, factor VII; FVII:C, factor VII plasma levels; IQR, interquartile range; rFVIIa, recombinant activated factor VII; TA, tranexamic acid.

### Selection of Periprocedural Hemostatic Treatment Depending on the Surgical Risk of Bleeding and the Severity of FVIID

**Fig. 1:**
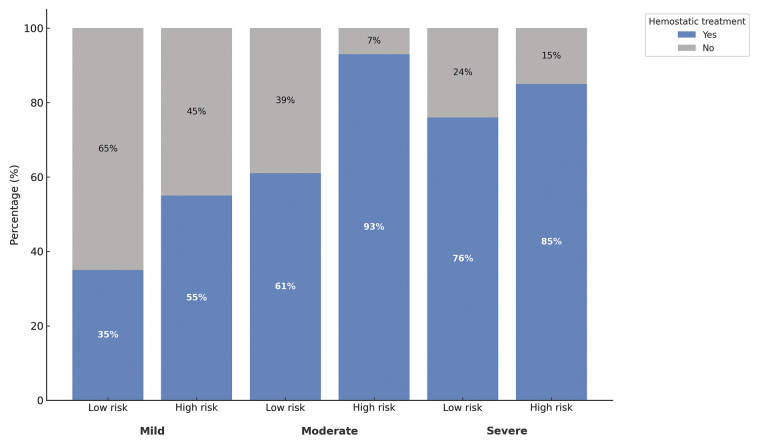
Hemostatic treatment based on factor VII deficiency (FVIID) severity and surgical bleeding risk. Severity of FVIID is represented on x-axis. Each severity subgroup was divided into low- and high-risk surgeries following ISTH recommendations. Low-moderate bleeding risk surgeries were represented as “low risk”.

**Fig. 2:**
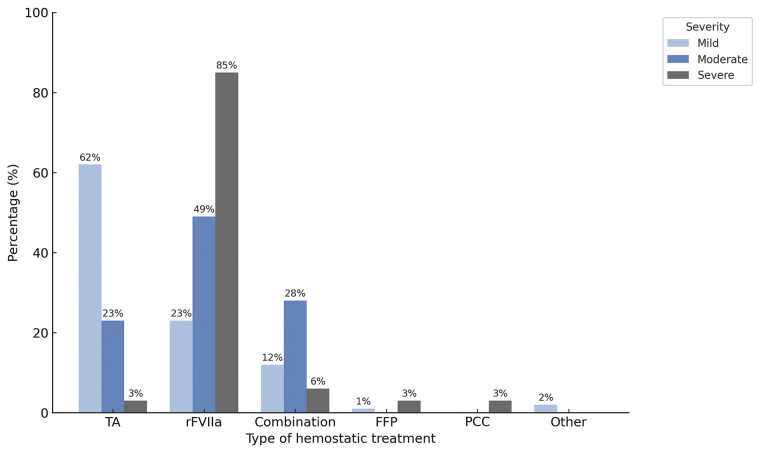
Type of hemostatic treatment depending on the severity of factor VII deficiency (FVIID). The percentages shown in the figure refer to the total number of procedures in which hemostatic treatment was used, depending on the severity of FVIID and regardless of the surgical risk. Mild = 119 surgeries, moderate = 35 surgeries, severe = 33 surgeries. FFP, fresh frozen plasma; PCC, prothrombin complex concentrate; rFVIIa, recombinant activated factor VII; TA, tranexamic acid.

When considering the bleeding risk associated with the procedure, hemostatic prophylaxis was administered in 42.8% of LR and 62.8% of HR surgeries. The most frequently used treatment for HR procedures was monotherapy with rFVIIa, which accounted for 43.5% of cases; this percentage increased to 60.6% when combined with TA. In contrast, for LR procedures, the most commonly used treatment was monotherapy with TA, which was utilized in 49.6% of cases. Other approaches, such as fresh frozen plasma (FFP) or prothrombin complex concentrate (PCC), were used in less than 2% of the procedures ( Table 1 and Supplementary Table S1 , available in the online version only). When considering both bleeding risk and severity of FVIID, in patients with mild FVIID, hemostatic treatment was administered in 35% (67/194) of LR and 55% (52/94) of HR surgeries, primarily utilizing TA monotherapy (62%). In patients with moderate and severe FVIID, hemostatic treatment was used in a higher percentage of surgeries, with 61 to 76% in LR and 93 to 85% in HR surgeries, respectively. The most commonly used treatment for moderate FVIID was rFVIIa, which was given in 67.5% of cases, either as monotherapy (42.5%) or in combination with TA (25%). For severe FVIID, rFVIIa was administered as monotherapy in 85% (28/33) of cases ( Figs. 1 and 2 , Supplementary Table S1 , available in the online version only). The duration of treatment with rFVIIa typically lasts from 1 to 3 days, administered every 4 to 6 hours based on the patient's bleeding risk and any periprocedural complications. The most common dosage of rFVIIa was between 15 and 30 µg/kg. In some cases, involving patients with mild or moderate FVIID, procedures were performed using a dosage of 10 µg/kg. The doses of TA administered varied considerably across centers, reflecting differences in clinical practice and the type of surgical procedure performed. For LR surgical procedures, the most common dosage was 500 mg every 8 hours on the first day, or until bleeding from the surgical site ceased. For HR procedures, 1 g was administered every 6 to 8 hours for 2 to 3 days or until bleeding stopped.

### Bleeding Outcomes and Hemostatic Coverage

The overall incidence of bleeding complications was 3.1% (12/380), and in 6 out of 12 procedures (50%) surgical bleeding was considered major according to ISTH criteria. In 11 out of 121 (9%) HR and 1 of 259 (0.4%) LR surgeries, a bleeding episode occurred, showing a significantly higher rate of complications in HR surgeries ( *p* = 0.01). All major bleeding cases requiring hemostatic treatment had good or excellent hemostatic coverage. In our analysis of subgroups, we found that 10 out of 288 patients (3.5%) with mild FVIID experienced bleeding complications. In the moderate deficiency group, 2 out of 50 patients (4%) had bleeding, while no patients in the severe deficiency group experienced bleeding ( Table 2 ). Among the patients with mild FVIID, 90% (9/10) of bleeding complications were observed during HR surgeries, and all these patients had a reported history of bleeding ( Table 3 ). More than half of them either did not receive any hemostatic treatment or were treated with TA in monotherapy. All except one major bleeding happened in the mild subgroup and had a BS ≥3. Additionally, 7 of 12 (58%) surgical complications were observed in HR procedures and had BS ≥3. In the only LR surgery, the bleeding episode was noted in a patient with mild FVIID and a prior history of bleeding, treated with TA monotherapy ( Table 3 ). There was only one thrombotic complication documented, which was observed in one severe FVIID patient with no prior history of bleeding (BS = 0) who received rFVIIa ( Table 3 ).

**Table 2:** Hemostatic outcomes according to factor VII plasma levels, bleeding risk of surgery, and periprocedural hemostatic treatment

Factor VII deficiency	Total surgeries	Perioperative hemostatic treatment	*N* (%)	BS, median (IQR)	Factor VII:C median (IU/dL) (IQR)	High bleeding risk surgery	Low bleeding risk surgery	Bleeding complication
Mild (>20 IU/dL)	288	Yes	119 (41%)	0 (0–2)	34 (29–43.8)	52	–	4
						–	67	1
		No	169 (59%)	0 (0–2)	36 (30–45)	42	–	5
						–	127	0
Moderate (10–20 IU/dL)	50	Yes	35 (70%)	1 (0–2)	15.8 (11–18)	13	–	2
						–	22	0
		No	15 (30%)	0 (0–3)	17.6 (17–19.1)	1	–	0
						–	14	0
Severe (<10 IU/dL)	42	Yes	33 (79%)	3 (1–7)	5.3 (2.4–7.7)	11	–	0
						–	22	0
		No	9 (21%)	2 (0–7)	4 (1.6–5.7)	2	–	0
						–	7	0

Abbreviation: BS, bleeding score.

**Table 3:** Patients and surgeries with hemorrhagic complications

Patient number	Sex	Age	FVII:C (IU/dL)	Level	Bleeding score	Risk of surgery	Location	Hemostatic treatment	Description
211	Female	43	12.7	Mod	2	High	Shoulder repair surgery	rFVIIa + TA	Excessive bleeding from surgical site not requiring hemostatic treatment
205	Male	84	17.6	Mod	0	High	Total hip replacement	rFVIIa	Post-surgical hematoma requiring hemostatic treatment with rFVIIa (major bleeding)
138	Male	24	21	Mild	3	High	Multiple tooth extraction	TA	Excessive bleeding from surgical site requiring hemostatic treatment with TA
146	Female	50	27	Mild	3	High	Lung resection by thoracotomy	rFVIIa + TA	Severe post-surgical bleeding requiring blood transfusion (major bleeding)
156	Female	71	27	Mild	3	High	Breast cancer tumorectomy	rFVIIa + TA	Post-surgical hematoma requiring drainage and not hemostatic treatment (major bleeding)
127	Male	82	27.6	Mild	5	High	Total knee replacement	No	Severe post-surgical bleeding requiring blood transfusion (major bleeding)
8	Female	12	28.3	Mild	3	High	Tonsillectomy	No	Excessive bleeding from surgical site requiring hemostatic treatment with TA
125	Male	34	29	Mild	4	Low-Mod	Vasectomy	TA	Intraoperative hemorrhage leading to hemodynamic instability (major bleeding)
190	Female	50	32	Mild	8	High	Cesarean	No	Excessive bleeding from surgical site requiring hemostatic treatment with TA
181	Male	20	36	Mild	5	High	Tonsillectomy	No	Severe bleeding requiring blood transfusion and rFVIIa (major bleeding)
56	Female	49	47.3	Mild	2	High	Kidney tumorectomy	rFVIIa	Excessive bleeding from surgical site not requiring hemostatic treatment
88	Female	10	52	Mild	1	High	Tonsillectomy	No	Excessive bleeding from surgical site not requiring hemostatic treatment

Abbreviations: FVII:C, factor VII plasma levels; Mod, moderate; rFVIIa, recombinant activated factor VII; TA, tranexamic acid.

### Key Factors in Decision-making on Hemostatic Treatment and Predicting Surgical Bleeding Risks

**Fig. 3:**
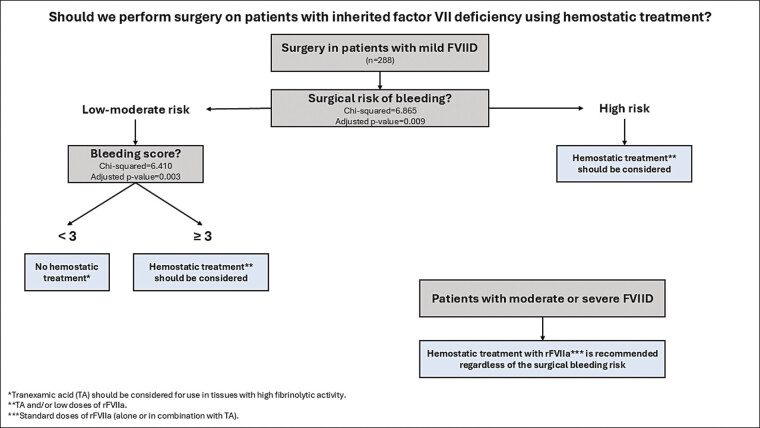
Decision-making tree to predict the use of hemostatic treatment. The decision-making tree simulation was based on data recorded. Levene's test indicated that variances were not homogeneous. We used the CHAID “tree growth” method to predict decision-making. Chi-squared quantifies the strength of correlation between independent variables and dependent ones (hemostatic treatment, Yes/No). The statistical significance of tests was supported by the *p* -value (of Chi-squared).

To make decisions about hemostatic treatment, the six variables mentioned above were studied. In the univariate analysis, we found that FVII plasma levels, BS, and the bleeding risk of surgery were significant parameters that influenced decision-making. However, FVII plasma levels and the bleeding risk of surgery emerged as the two key factors with the greatest impact on the decision process ( Table 4 ). We also assessed the predictive factors for bleeding complications using the same six variables. The BS and surgical risk were statistically significant parameters related to surgical bleeding. Among these, the surgical risk was found to have the strongest association with these complications. Further analysis by subgroups revealed that the surgical bleeding risk and BS were the strongest predictive factors for surgical bleeding in patients with mild FVIID ( Table 5 ). Based on the recorded data, we developed a statistical decision-making tree to predict the use of hemostatic treatment. This prediction is informed by the bleeding risk associated with surgery, the severity of FVII deficiency, and the BS. The algorithm recommends hemostatic treatment in mild FVIID patients for HR surgeries and LR procedures when the bleeding score is 3 or higher. Hemostatic treatment with rFVIIa is recommended in moderate and severe FVIID patients regardless of the surgical bleeding risk. Additionally, this algorithm effectively classifies 60 to 65% of cases, as demonstrated through various cross-validation methods applied to the sample ( Fig. 3 ).

**Table 4:** Univariate and multivariate analysis of possible factors influencing decision-making on hemostatic treatment

**(A) Univariate analysis**				
	***p*** **-value**	**Exp (B)/Odds ratio**	**95% CI for Exp (B) (min–max)**	**Wald**
FVII:C	<0.001	0.950	(0.934–0.965)	37.984
Risk of surgery	0.009	1.821	(1.161–2.856)	6.806
Bleeding score	0.001	1.163	(1.063–1.273)	10.760
Site of surgery	0.534	0.984	(0.936–1.035)	0.386
Sex	0.087	0.703	(0.469–1.053)	2.924
Age at surgery	0.247	0.995	(0.985–1.004)	1.338
**(B) Multivariate analysis**				
	***p*** **-value**	**Exp (B)/Odds ratio**	**95% CI for Exp (B) (min–max)**	**Wald**
FVII:C	<0.001	0.953	(0.937–0.970)	29.392
Risk of surgery	0.016	1.808	(1.119–2.919)	5.856
Bleeding score	0.109	1.084	(0.982–1.195)	2.570

Note: Statistical analysis was designed to stratify the weight of each significant variable in the decision to administer hemostatic treatment. Wald statistics classify FVII plasma levels as the most significant parameter in decision-making. We observe that BS did not reach *p* <0.05. Exp(B) = Exponent B, equivalent to odds ratio (OR), indicates inverse (<1) or direct (>1) relation with the dependent variable; CI = confidence interval; Wald = statistical value used to quantify the significance of the correlation with the dependent variable, inversely proportional to the *p* -value. FVII:C = factor VII plasma levels.

**Table 5:** Univariate and multivariate analysis of possible predictive factors of surgical bleeding in patients with mild factor VII deficiency

**(A) Univariate analysis**				
	***p*** **-value**	**Exp (B)/Odds ratio**	**95% CI for Exp (B) (min–max)**	**Wald**
Risk of surgery	0.009	16.020	(2.000–128.290)	6.829
Bleeding score	0.001	1.350	(1.122–1.623)	10.144
Site of surgery	0.886	0.988	(0.840–1.162)	0.020
Sex	0.494	0.638	(0.176–2.312)	0.467
Age at surgery	0.394	1.012	(0.985–1.039)	0.726
**(B) Multivariate analysis**				
	***p*** **-value**	**Exp (B)/Odds ratio**	**95% CI for Exp (B) (min–max)**	**Wald**
Risk of surgery	0.009	16.270	(1.982–122.529)	6.745
Bleeding score	0.003	1.346	(1.107–1.636)	8.907

Note: Statistical analysis was designed to stratify the potential weight of each significant variable as a predictive factor of surgical bleeding. Wald statistics classify the risk of surgery as the most significant parameter related to surgical bleeding. Exp(B) = Exponent B, equivalent to odds ratio (OR), indicates inverse (<1) or direct (>1) relation with the dependent variable; CI = confidence interval; Wald = statistical value used to quantify the significance of the correlation with the dependent variable, inversely proportional to the *p* -value.

## Discussion

Managing hemostasis in patients with FVIID during surgical procedures presents challenges due to the weak clinical–biological correlation between laboratory plasma factor VII:C levels and the risk of bleeding. Additionally, there are no established protocols or national or international recommendations to guide treatment, and recommendations are usually based on biological parameters. 25 Our study showed that despite the poor correlation between FVII:C and bleeding phenotype, the plasma levels of FVII are usually used to select the hemostatic treatment. In our cohort, we also found a weak association between factor VII:C and the BS, which aligns with findings from the EN-RBD network in 2012. 15 This poor or absence of correlation was particularly evident in patients with mild FVIID, while a moderate correlation was observed in those with severe FVIID, similar to the results reported in the Rare Bleeding Disorders in the Netherlands (RBiN) study in 2020. 14 Despite this, FVII:C levels often play a central role in therapeutic decisions. 25 However, data from the STER registry and other studies suggest that a patient's bleeding history may be a more relevant factor in guiding treatment, especially in severe cases of FVIID. 32 33 34 Notably, the choice of treatment based on clinical criteria in the STER registry was primarily representative of the severe FVIID subgroup. Our data indicated that the decision to administer hemostatic treatment was primarily driven by the severity of FVIID and by the risk of surgical bleeding ( Table 4 ). Consequently, treatment was more frequently used in patients with lower FVII:C levels and those undergoing HR procedures. Additionally, the choice of therapeutic agent (TA vs. rFVIIa) was also influenced by these factors; TA was preferred for mild cases of FVIID or LR surgeries, and rFVIIa was used in patients with severe FVIID or HR surgeries. Consistent with our findings, most authors recommend rFVIIa as the treatment of choice, typically administered at 15 to 30 µg/kg every 4 to 6 hours until bleeding stops or for 24 to 72 hours postoperatively. 35 36 37 38 Recent studies suggest that using doses lower than the labeled amount may still provide sufficient hemostatic results while also reducing the risk of thrombosis and lowering treatment costs. 39 40 Moreover, we found that the BS and the associated risk of bleeding from the procedure were the main predictors of surgical bleeding, particularly for patients with mild FVII deficiency ( Table 5 ). Identification of bleeding phenotype as a predictor of surgical bleeding has been reported by other authors, which highlights the importance of this clinical variable as one of the central axes in periprocedural management decision-making. 33 34 The overall surgical bleeding rate in our study (3.1%) is comparable to that reported in other registries such as STER (4.5%) or the French MARACHI study (6.1%). 32 33 34 35 36 37 The significantly higher rate observed in RBiN probably reflects its focus on dental procedures, most of which did not cause major bleeding or require hemostatic treatment. 14 41 In our study, we observed that most documented bleeding complications, including major bleeding, occurred in patients with mild FVIID and a BS of 3 or higher, indicating that the bleeding phenotype may offer additional insight for risk stratification beyond FVII:C levels. A notable finding is the absence of bleeding complications in patients with severe FVIID. In contrast, postoperative bleeding episodes were common among individuals with mild deficiency. This paradox can be attributed primarily to differences in treatment approaches based on the severity of FVIID. Notably, the majority of patients with severe deficiency received prophylactic hemostatic treatment, particularly rFVIIa, even for LR procedures. In comparison, fewer than half of the patients with mild deficiency received treatment, and when they did, it was often limited to TA alone ( Table 2 , Fig. 2 ). Consequently, analyses of surgical bleeding predictors (both univariate and multivariate) were specifically conducted on the subgroup of patients with mild deficiency. As a result, the absence of bleeding events in the severe deficiency group likely reflects the effectiveness of prophylactic treatment rather than a lower intrinsic bleeding tendency. Additionally, several bleeding events happened during HR surgeries, underscoring that the surgical context is a key factor in determining bleeding risk. These findings emphasize the necessity of considering both bleeding history and the nature of the surgical procedure when making decisions about prophylactic treatment, especially in patients with mild deficiency. A tailored approach to replacement therapy based on clinical phenotype and surgical risk may help optimize the balance between efficacy and safety ( Fig. 3 ). It is also important to note that a thrombotic complication documented in this study occurred in a severe FVIID patient with a BS of 0 following prolonged immobilization surgery (hip replacement) and the use of periprocedural rFVIIa. Although the number of patients and surgeries included in our study is comparable to other reports on FVIID, 25 32 there are several limitations to consider: (a) data collection and decision-making simulations were based on retrospective information, using a statistical tool intended to replicate real-life decision-making, which may introduce bias; (b) the presence of varying management strategies across different centers may have resulted in a lack of standardization in peri-surgical hemostatic protocols; (c) the inclusion of a wide range of surgical procedures within both LR and HR categories introduces anatomical and technical heterogeneity, potentially influencing bleeding risk and the need for prophylactic treatment; (d) most patients with severe FVIID received hemostatic replacement therapy, which effectively prevented bleeding. Consequently, the lack of statistical significance of FVII:C levels as a predictor of bleeding likely may reflect treatment effects rather than true clinical risk, limiting the interpretability of this variable; (e) monitoring thrombotic complications at the 30-day mark proved complex in certain cases because some patients had surgeries at centers other than their reference center, or due to variations in the use of thromboprophylaxis. It is important to note that FVII:C levels were measured using recombinant human thromboplastin, which may have prevented the marked underestimation observed with rabbit thromboplastin in patients with certain mutations, such as the Padua variant. 42 Finally, when interpreting the decision algorithm, it's important to remember that most procedures are performed on patients with mild FVIID (75.8%). These patients tend to have a weaker or no correlation between FVII:C levels and their bleeding scores. To make our simulations more reflective of routine clinical practice, we should prioritize standardizing management protocols and prospective data collection. Our findings support the following approach for patients with mild FVIID: (A) for HR surgeries, hemostatic treatment with TA and/or low doses of rFVIIa should be considered. However, as reported by other authors, select HR procedures may be safely performed without replacement therapy in patients with mild FVIID and no prior bleeding history (BS = 0). 25 (B) For LR procedures, treatment decisions should be individualized according to the patient's bleeding phenotype. In those with a bleeding tendency (BS ≥ 3), hemostatic therapy should be considered, preferably with TA and/or low-dose rFVIIa ( Fig. 3 ). (C) Complex cases, such as LR surgery in elderly patients with a history of both bleeding and thrombosis, require individualized decision-making, where the use of low-dose rFVIIa could be considered. (D) In procedures involving areas with high fibrinolytic activity, TA either alone or in combination may be appropriate. (E) For patients with BS <3 undergoing surgery in anatomically challenging areas, a tailored approach is necessary, taking into account their comorbidities and past bleeding history. Additionally, for patients with moderate or severe FVIID, we recommend hemostatic treatment with standard doses of rFVIIa regardless of the surgical bleeding risk. The recommended rFVIIa doses in the algorithm are based on clinical practice observed in our study. Further prospective research is required to optimize these doses and define them more specifically for each case.

## Conclusion

This study highlights the relevance of both plasma FVII levels and surgical risk in guiding current hemostatic management. However, the increased rate of bleeding complications in patients with mild FVIID and higher bleeding scores who did not receive hemostatic treatment emphasizes the critical role of bleeding phenotype in clinical decision-making.

## Notes

**What is known about this topic?**

- Patients with inherited factor VII deficiency (FVIID) exhibit a wide range of bleeding symptoms.
- Surgery for FVIID is challenging due to the lack of standardized protocols and the limited evidence on bleeding predictors.

**What does this paper add?**

- Despite the poor correlation between FVII plasma levels and bleeding phenotype, it is usually used to determine the hemostatic treatment.
- The bleeding score and the associated risk of bleeding from the procedure are crucial factors in deciding on hemostatic treatment, particularly for patients with mild FVII deficiency.
