# Supplementary material for: Optimizing Surgical Approaches for Patients with Inherited Factor VII Deficiency
Source: Thromb Haemost. 2026 Jan 6;126(8):809–18. doi: 10.1055/a-2778-4440 (PMC13421291; doi:10.1055/a-2778-4440)
Supplement: Supplementary file 1 — Supplementary Material [file 10-1055-a-2778-4440_29249654.pdf]

Supplementary Table 1. Peri-procedural hemostatic treatment used according to the severity of FVIID and the bleeding risk of surgery.

FVII deficiency and total surgeries, n (%)	*Risk bleeding surgery*	*Hemostatic treatment (Yes/No)*	*Type of treatment*
Mild = 288 (75.8%)	Low risk = 194	Hemostatic treatment = 67 (35%)<br>No hemostatic treatment = 127 (65%)	Tranexamic acid = 47 (70.1%)<br>rFVIIa = 12 (17.9%)<br>Combination = 6 (9%)<br>Fresh frozen plasma = 1 (1.5%)<br>Other = 1 (1.5%)
	High risk = 94	Hemostatic treatment = 52 (55%)<br>No hemostatic treatment = 42 (45%)	Tranexamic acid = 27 (52%)<br>rFVIIa = 16 (30.7%)<br>Combination = 8 (15.4%)<br>Other = 1 (1.9%)
Moderate = 50 (13.2%)	Low risk = 36	Hemostatic treatment = 22 (61%)<br>No hemostatic treatment = 14 (39%)	rFVIIa = 9 (40.9%)<br>Tranexamic acid = 7 (31.8%)<br>Combination = 6 (27.3%)
	High risk = 14	Hemostatic treatment = 13 (93%)<br>No hemostatic treatment = 1 (7%)	rFVIIa = 8 (61.5%)<br>Combination = 4 (30.8%)<br>Tranexamic acid = 1 (7.7%)

Thrombosis and Haemostasis | DOI 10.1055/a-2778-4440 | © Thieme. All rights reserved.

Severe = 42 (11%)	Low risk = 29	Hemostatic treatment = 22 (76%)<br><br>No hemostatic treatment = 7 (14%)	rFVIIa = 19 (86.5%)<br>Tranexamic acid = 1 (4.5%)<br>Combination = 1 (4.5%)<br>Fresh frozen plasma = 1 (4.5%)
	High risk = 13	Hemostatic treatment = 11 (85%)<br><br>No hemostatic treatment = 2 (15%)	rFVIIa = 9 (82%)<br>PCC = 1 (9%)<br>Combination = 1 (9%)

Abbreviatures: rFVIIa = recombinant activated factor VII. PCC = prothrombin complex concentrate. Combination = Tranexamic acid + rFVIIa.

Thrombosis and Haemostasis | DOI 10.1055/a-2778-4440 | © Thieme. All rights reserved.
